# Older Adults’ Perceptions of Psychotherapy in Cyprus

**DOI:** 10.3390/bs9110116

**Published:** 2019-11-19

**Authors:** Ioanna Katsounari

**Affiliations:** Department of Psychology and Social Sciences, Frederick University Cyprus, Nicosia 2334, Cyprus; soc.ki@frederick.ac.cy

**Keywords:** psychotherapy, elderly, Cyprus

## Abstract

The purpose of the study was to explore older adults’ views and perceptions of psychotherapy in Cyprus. A total of 25 older adults, aged between 65–89, participated in semi-structured interviews. Thematic analysis identified three main themes: Familiar term/Unfamiliar process; existential crises during this stage; and the issue of stigma in psychotherapy. Participants indicated a basic understanding of what psychotherapy entails, but did not associate psychotherapy with serious mental illness. Participants identified a number of existential issues that are potentially major life stressors for an elderly person and referred to the historical stigma that has an impact on their own perceptions about psychotherapy. However, participants seemed to view their ability to overcome psychological difficulties on their own as a proof of personal strength. Psychologists and other health professionals also need to be mindful of how they describe psychological concepts and treatment, as older adults may not understand what they are being told or may be afraid of what treatment involves based on historical context. This study highlights the importance of using strategies that may have the potential to empower this population in order to proactively attend to their mental health, including community-based education and national mental health campaigns.

## 1. Introduction

The percentage of the population in Cyprus over the age of 65 was 13.9 per cent last year while 3.1 per cent were over the age of 80, figures that will rise to 25.2 per cent and 10.8 per cent, respectively, by 2080. Cypriot women who reach the age of 65 can expect to live another 21.6 years on average while Cypriot men will live another 18.6 years on average once they reach that age [1].

In Cyprus there are no detailed data on the prevalence of psychiatric disorders among the elderly. According to World Health Organization (WHO) statistics, depression is the most common mental health problem in this age group with estimated prevalence rates of 22% for men and 28% for women aged 65 or over and 40% of older people in care homes. Anxiety disorders affect 1 in 20 older people. Less commonly, elderly patients present to services with psychosis due to bipolar disorder or a psychotic disorder [2].

Older individuals may experience life stressors common to all people, but also stressors that are unique or more common in later life. Older adults may experience reduced mobility, chronic pain, and health problems. In addition, they are more likely to experience events such as loss, bereavement, and a drop in socioeconomic status. All these stressors can result in isolation, loneliness, or psychological distress in older people [3]. Older adults with physical health conditions such as heart disease have higher rates of depression than those who are healthy [4]. Furthermore, elder abuse is now recognized internationally as an extensive and serious problem. Older adults are vulnerable to physical, verbal, psychological, financial, and sexual abuse; abandonment; neglect; and subsequent losses of dignity and respect. Elder abuse can lead not only to physical injuries, but also to serious, sometimes long-lasting psychological consequences, including depression and anxiety [5]. Although reported rates of mental illness are lower than younger adults, suicide rates are often higher among older adults [6]. 

Despite this high prevalence of mental disorders among older persons, this cohort’s access to mental health services is lower than its rate of need, and has grown only modestly in recent years [7]. Most community-dwelling older adults continue to seek assistance for mental health problems from primary care physicians, who have repeatedly demonstrated inadequate ability to recognize disorders such as depression or to provide appropriate referrals [7]. Researchers have identified many barriers that contribute to poor access and inadequate use of mental health services by older adults including lack of perceived need for care [8], lack of knowledge about availability of mental health services [9], stigma [10], negative professional attitudes about working with the elderly [11], limited availability of affordable services [12,13], low rates of referral by general practitioners [14], and difficulty arranging transportation [12]. 

In particular, attitudes towards mental health services by older adults have been suspected as major barriers to seeking treatment. Reasons why older adults have been thought to reject mental health care include lack of education regarding mental health and generational negative attitudes or stigma surrounding mental illness [15]. It has been proposed that older adults tend to associate mental disorders with personal failure or spiritual deficiency [16]. However, other research found that older participants believed access to mental health services was important despite being unlikely to access the services themselves [12,13].

There is an increasing recognition that patients’ views of mental health treatments are an important factor to consider in the effort to increase utilization of services [17]. Previous research assessing older peoples’ perceptions and views of the nature and purpose of psychotherapy is limited. Research by reference [18] explored older peoples’ attitudes towards, and understanding of, psychotherapy and their willingness to seek out psychological services for themselves. Most participants were found to be reluctant to disclose emotional concerns to their doctor and GPs (General Practitioners) were seen as responsible for initiating discussions about mental health. Lack of enquiry by GPs, short consultation times and unfamiliarity with psychological terminology were considered barriers for accessing treatment. Participants mostly held positive and accepting views of psychotherapy but a lingering stigma, based on historical values of stoicism and self-reliance, was evident. Reductions in societal stigma were attributed to increased exposure to mental health information via the media, however, participants appeared to confuse psychological services with counselling as this was a term that most were familiar with.

This study aimed to use a qualitative approach to explore older adults’ perceptions of psychotherapy in Cyprus. One of the most distinctive features of qualitative research is that this approach allows one to identify issues from a participant’s point of view, and understands the meaning and interpretations participants give to behaviors, events, or objects [19]. A qualitative methodology was deemed appropriate for the purposes of this research as it allows for in-depth exploration of the subject under study. This is the first study conducted in Cyprus exploring older adults’ perceptions of the process of psychotherapy as well as their views on the reasons for seeking psychological help, and ways that psychotherapy can help with life stressors during this stage of life. It has to be noted that psychotherapy as a profession is popular in Cyprus despite the fact that psychotherapy training takes around 3–4 years. Psychotherapy can be provided by psychologists (clinical) but also other professionals trained in the various psychotherapy schools.

## 2. Materials and Methods

This study comprised 25 participants aged between 65 and 89 years (M = 79.5; SD = 6.64), 15 women and 10 men. A convenience sample was used and the selection of interviewees was based on the following inclusion criteria: (1) to be over 65 years of age, and (2) to be in a good mental condition. Interviewees were visitors to a community center which offers recreational activities for older individuals. Efforts were made to ensure that both genders were represented. Only three participants reported that they had previously attended psychotherapy sessions, while two participants reported using psychiatric medication for depression and anxiety, which was prescribed through a psychiatrist/neurologist.

For data collection, the researcher constructed a semi-structured interview comprising of six open-ended questions regarding the participants’ perceptions around the process of psychotherapy, the reasons an older adult may attend psychotherapy, the benefits that an older adult may derive from psychotherapy, as well as their knowledge in regards to the manner that one can access psychological help, and their experience accessing psychological help (if relevant). Interviews ranged from 30 to 60 min in duration and were digitally recorded. Potentially identifying information was removed.

The data was analyzed with the use of thematic analysis based on the guidelines outlined by Braun and Clarke [20]. Thematic analysis allows the researcher to identify ‘repeated patterns of meaning’ [21] within research data and provides a method for describing and interpreting emergent themes. For this purpose, transcribed interviews were read several times. Interviews were coded for common themes or patterns. Following this, the initial codes were put in different themes and sub-themes. All themes were named and selected data extracts that reflected the essence of participants’ views regarding psychotherapy were identified.

## 3. Results

The thematic analysis revealed three themes: familiar term/unfamiliar process, existential crises during this stage, and the issue of stigma in psychotherapy. The three themes are overlapping and some extracts contain references to more than one theme.

### 3.1. Theme 1: Familiar Term/Unfamiliar Process

Most participants were familiar with the term “psychotherapy” and also recognized the basic processes involved in psychotherapy. Most participants identified that it involves ‘treating the soul’ (psyche). Some participants explained that their understanding of psychotherapy relates to expressing thoughts that could not be processed by one alone. Participants described psychotherapy as a ‘problem-solving’ process and the role of the psychotherapist with older adults as one of giving direct advice ‘for resolving psychological problems that one cannot resolve alone’. It appeared from the participants’ disclosures that this is a familiar terminology to them; it was linked with the term “counselling”. Interestingly, most participants referred to the cognitive aspect of treatment as opposed to receiving help for emotional difficulties. As one participant stated, ‘Psychotherapy is the science which helps us by analyzing the problem and providing correct solutions. Every person is different, therefore, in every person it helps to resolve different issues’. Therefore, most participants did not associate psychological terminology with serious mental health problems and disturbing treatment procedures. 

Among those with previous experience with psychotherapy, a greater level of familiarity with terminology the processes involved in psychotherapy was evident: ‘Psychotherapy is the therapeutic process during which mental disorders are treated. It is conducted through a conversation in regular meetings, between a qualified psychotherapist and the person with a mental disorder’. Another participant stated, ‘Psychotherapy is a process through which a person’s functionality towards its family and society in general is improved’.

Despite the fact that most participants showed familiarity with the concept of psychotherapy and were able to articulate ideas articulated a basic knowledge of what psychotherapy involves, when asked whether they would seek psychological help when confronted with a serious issue, they expressed that in the past they used their own coping mechanisms instead of seeking professional help. In particular, participants seemed to view their ability to overcome personal difficulties on their own as a sign of personal strength. 

In addition, when asked to elaborate on ways to access psychological help, most participants were familiar with the process. Few participants suggested that access to a psychotherapist occurs through a general practitioner (GP), while most participants expressed favorable opinions about the recent implementation of the General Healthcare System in Cyprus. General practitioner is a term used in Cyprus to denote a physician, through which referrals are made to specialist doctors. In particular, most participants expressed that the new Healthcare system carries many benefits such as: access to qualified psychologists, quick access to services, and provision of low cost services.

Two participants described receiving psychiatric medication in the past in order to cope with personal difficulties. These participants appeared to have difficulty differentiating the role of different mental health professionals (e.g., psychiatrist, neurologist, psychologist). They also did not seem to realize that these difficulties could be resolved by attending psychotherapy. Furthermore, only one participant appeared to be aware of the different applied specializations within psychology practice, the different levels of trainings required, or the registration requirements for psychologists. 

### 3.2. Theme 2: Existential Crises during This Stage

Participants identified both psychological and physical causes as the potential reasons for seeking psychological help at later age. All participants identified depression as the main reason that an older person would attend psychotherapy. In addition, all participants identified loneliness as a major life stressor for older adults, especially after the loss of a spouse and children living away from home. As one participant stated, ‘This is especially a difficult period, when older people live alone, they cannot find the strength to overcome this, especially women, because they were used to take care of everyone in the family’. Some participants expressed that loneliness is even more difficult to overcome when relations with children are conflictual: ‘After years of growing up children together, it is really hard to lose your spouse. When you feel rejected by your own children, it is even harder’. Participants identified conflicts within the family system in general as a major life stress at an older age. 

In addition, most participants identified that older adults’ isolation can have secondary effects such as depression, lack of self-esteem, and anxiety. Most participants recognized that economic issues can also lead to isolation. More specifically, lack of adequate funds can restrict an individual’s available options for outings and entertainment, thus contributing further to their isolation. Emotional difficulties according to these participants, are a direct consequence of the psychosocial changes that are prominent at an older age. 

Participants identified a variety of other existential issues that can function as life stressors for the elderly. The experience of loss in the person’s later life and the reality of death were the most common identified issues. Participants expressed that multiple losses suffered during an older person’s lifetime can have an impact on a person’s outlook of life. As one participant stated, ‘To experience loss and death, inevitably brings a change of perspective in the person, in the way that he/she perceives the world and in the way he/she interprets it’. The participants which discussed the fear of death also stressed the need of psychotherapy for older adults, in order to plan for the future ahead. In general, participants expressed the fact that older individuals may experience existential crises during which they re-evaluate their past life. They noted the need for psychotherapy in order to help the person process past life achievements and failures and to accept the present realities.

Physical ailment can be another source of stress for the elderly. Participants discussed the fact that confronting serious illnesses (e.g., Alzheimer’s disease) can be a challenge for an older person. Specifically, participants appeared to associate this with becoming a burden on loved ones. Therefore, the need to rely on others in order to meet one’s needs can be particularly challenging for older people and an area that psychotherapy can help by challenging these assumptions and providing support for the older person. As one participant stated, ‘They really need to come into terms with their present physical and socioeconomic reality. They need to accept that human nature imposes limits on them. And that these are different limits from the ones that society imposed on them.’

### 3.3. Theme 3: The Issue of Stigma in Psychotherapy

Almost all participants agreed that societal stigma, related to seeking professional psychological help, has been reduced significantly compared to the past. At the same time, participants discussed the fact that it is still quite rare for a person in their generation to attend psychotherapy for psychological difficulties. This line of thought is related to a culture of self-sufficiency and stoicism endorsed by older generations and transmitted as a cultural value. Participants described that in the past, the prejudice which existed against mental illness and its treatment had a strong impact on their own perceptions of psychotherapy. One participant recalled: ‘In the past, whoever had a mental illness was ostracized by society. The entire family was secluded from the social context. The person with the mental illness was hidden as someone who had committed a crime. The church had an impact on stigmatizing views as it was believed that the person with a mental illness or the family had sinned’. Furthermore, the historical association between mental illness and commitment in a mental health hospital also contributes to the fear and stigma that older individuals have around seeking help for mental health issues. A few participants explained that the media’s negative portrayal of mental illness has not been helpful in reducing stigma.

Most participants expressed that they were raised with the value that being able to cope with life’s difficulties and psychological suffering is an indication of personal strength. Another value was that disclosing personal emotions and thoughts to an outsider of the family is considered inappropriate. These values appear to continue to have a negative impact on treatment-seeking behavior. ‘In the past we did not go to see a psychologist, if you had a problem, it was discussed within the family. I remember my mother suffering from post-partum depression after giving birth to my sister. Nobody even considered referring her to a professional for support, it was considered that she had to endure and resolve this on her own’. 

Despite the fact that participants in general discussed the societal stigma that predominated in the past, they also agreed that there has been a change in society’s attitudes. Participants in this study also agreed that they view this as a positive change. However, despite the fact that all participants described psychological difficulties in the past years, only two participants reported that they had sought professional help. In addition, when asked whether they would attend psychotherapy for future difficulties, more than half of the participants expressed that they would likely resolve any issue on their own. This attitude was prevalent in individuals over the age of 70. It appears that feelings of stoicism and self-sufficiency when it comes to emotional difficulties are still lingering in older adults, especially those over the age of 70.

## 4. Discussion

The aim of this study was to explore older adults’ perceptions about psychotherapy. Three general themes emerged as a result of the thematic analysis, *(a) Familiar term/unfamiliar process; (b) existential crises during this stage; and (c) the issue of stigma.* It was evident during the presentation of the findings that these three themes overlap and are interconnected to a large extent.

Almost all participants indicated a basic understanding of what psychotherapy entails. Most participants described psychotherapy as a problem-solving process which provides solutions to difficult situations. Very few participants expressed that psychotherapy addresses the emotional aspect of psychological dysphoria. The fact that almost all participants did not associate psychotherapy with serious mental illness, shows that they appear to confuse psychotherapy with counseling. This study highlights a need for efforts to increase mental health literacy among Cypriot older adults. Research has indicated that older adults exhibit poorer mental health literacy than younger adults, including less accuracy at identifying symptoms of mental disorders, and endorsing fewer sources of treatment for a mental disorder [22,23,24]. It has been proposed that older adults in closer proximity to someone with a mental disorder are more likely to have better mental health literacy, a finding that has the potential to inform mental health education and promotion strategies in this population [22]. Other research suggests that older adults might get more out of mental health literacy programs in group or social settings. Programs that use older adult peer educators/supporters, such as the "older people’s champions" of the Healthy Passport program in England, might make the programs more effective. Mental health campaigns, such as Australia’s beyondblue, might increase the mental health literacy of older adults [17,24]. In Cyprus, where stigma around mental illness holds a long tradition and persists among the elderly, it is important to use strategies that have the potential to empower this population to proactively attend to their mental health including community-based education and national mental health campaigns.

Participants identified a number of existential issues that are potentially major life stressors for an elderly person, such as loneliness, isolation, fear of death, coping with multiple losses, managing conflict within the family system, coping with limited socioeconomic resources, and coping with ageing and failing health. They acknowledged that these issues can cause secondary difficulties such as depression, lowered self-esteem, anxiety, and fear. Despite the fact that all participants expressed that psychotherapy can help resolve these issues, most participants did not seem to personally relate to this view. Instead, they expressed the view that one should endure and resolve emotional difficulties on their own. This perception appears to be ingrained through past generations in Cyprus that mental illness is a personal issue that needs to be kept and resolved within the family. It is also associated to the view that not managing to effectively deal with emotional difficulties is a sign of personal weakness. The belief of self-reliance for addressing mental and emotional issues has been indicated in other research [18,23]. Despite the fact that participants’ expressed positive views in regards to the decreased stigmatization of mental illness in Cyprus, a tendency to refer to mental illness as something that involves ‘others’ was also evident through participants’ discourse. These findings stress the importance of the role of GPs as the first points of contact for an older person. In particular, GPs need to be aware that older adults may not spontaneously disclose emotional and psychosocial problems and that some believe that disclosing about these issues is a sign of personal weakness. Therefore, GPs should be mindful to inquire about these issues in medical consultations and refer to psychologists when appropriate. Psychologists and other health professionals also need to be mindful of how they describe psychological concepts and treatment as older adults may not understand what they are being told or may be afraid of what treatment involves based on historical context [18,22,24].

It is important to acknowledge that this study was a small, qualitative study of 25 older adults. Consequently, it is not possible to generalize the findings to all older adults in Cyprus. Despite the aforementioned limitation, the findings drawn in this study resonate with previous literature regarding older peoples’ perceptions of psychotherapy and they raise important issues regarding access to these services. Findings suggest that older adults are generally not aware of services provided by psychologists in Cyprus, the different levels of training required (e.g., for Clinical Psychologists versus Psychotherapists), or the registration regulations for psychologists in Cyprus. As such, this study has indicated that initiatives need to be taken in order to increase older adults’ knowledge of the psychology profession as well as willingness to seek professional mental health treatment, if needed. This is deemed an urgent matter, as worsening socioeconomic circumstances in Cyprus will affect a greater number of elderly with a subsequent impact on their mental health. 
